# p62/SQSTM1/Keap1/NRF2 Axis Reduces Cancer Cells Death-Sensitivity in Response to Zn(II)–Curcumin Complex

**DOI:** 10.3390/biom11030348

**Published:** 2021-02-25

**Authors:** Alessia Garufi, Eugenia Giorno, Maria Saveria Gilardini Montani, Giuseppa Pistritto, Alessandra Crispini, Mara Cirone, Gabriella D’Orazi

**Affiliations:** 1 Unit of Cellular Networks, Department of Research and Advanced Technologies, IRCCS Regina Elena National Cancer Institute, 00144 Rome, Italy; alessiagufi@ifo.gov.it; 2 School of Medicine, University “G. D’Annunzio”, 66013 Chieti, Italy; 3 Laboratory MAT_IN LAB, Department of Chemistry and Chemical Technologies, Calabria University, 87036 Rende, Italy; eugenia.giorno@unical.it (E.G.); alessandra.crispini@unical.it (A.C.); 4 Department of Experimental Medicine, Sapienza University of Rome, Laboratory Affiliated to Pasteur Institute Italy Foundation Cenci Bolognetti, 00185 Rome, Italy; mariasaveria.gilardinimontani@uniroma1.it (M.S.G.M.); mara.cirone@uniroma1.it (M.C.); 5 Centralized Procedures Office, Italian Medicines Agency (AIFA), 00187 Rome, Italy; pistritto@uniroma2.it; 6 Department of Neurosciences, Imaging and Clinical Sciences, University “G. D’Annunzio”, 66013 Chieti, Italy

**Keywords:** p62/SQSTM1, Keap1, NRF2, cancer cells, p53, zinc compounds, drug sensitivity

## Abstract

The hyperactivation of nuclear factor erythroid 2 p45-related factor 2 (NRF2), frequently found in many tumor types, can be responsible for cancer resistance to therapies and poor patient prognosis. Curcumin has been shown to activate NRF2 that has cytotprotective or protumorigenic roles according to tumor stage. The present study aimed at investigating whether the zinc–curcumin Zn(II)–curc compound, which we previously showed to display anticancer effects through multiple mechanisms, could induce NRF2 activation and to explore the underlying molecular mechanisms. Biochemical studies showed that Zn(II)–curc treatment increased the NRF2 protein levels along with its targets, heme oxygenase-1 (HO-1) and p62/SQSTM1, while markedly reduced the levels of Keap1 (Kelch-like ECH-associated protein 1), the NRF2 inhibitor, in the cancer cell lines analyzed. The silencing of either NRF2 or p62/SQSTM1 with specific siRNA demonstrated the crosstalk between the two molecules and that the knockdown of either molecule increased the cancer cell sensitivity to Zn(II)–curc-induced cell death. This suggests that the crosstalk between p62/SQSTM1 and NRF2 could be therapeutically exploited to increase cancer patient response to therapies.

## 1. Introduction

Nuclear factor erythroid 2 p45-related factor 2 (NRF2) is the master regulator of oxidative stress and is often upregulated in solid cancers, promoting proliferation and resistance to chemotherapy and to apoptosis [1,2]. NRF2 activation can be achieved either in a canonical or in a non-canonical way. Under normal conditions, NRF2 is continuously inactivated by ubiquitin-proteasome degradation following its interaction with Kelch-like ECH-associated protein 1 (Keap1)-associated E3 ubiquitine ligase [3]. In canonical NRF2 activation, which is under oxidative or electrophilic stress, the Keap1 ubiquitine ligase activity declines, leading to the stabilization of NRF2 that subsequently translocates to the nucleus where it induces the transcription of antioxidant response element (ARE)-bearing target genes, including heme oxygenase-1 (HO-1), NAD(P)H quinone oxidoreductase (NQO1) and glutathione (GSH). [3]. In non-canonical NRF2 activation, p62/sequestome 1 (SQSTM1, hereafter referred as p62), a key autophagyc adaptor, interacts with Keap1, inducing its degradation through autophagy, thereby triggering NRF2 stabilization and activation [4]. On the other hand, NRF2 can induce p62 transcription by binding antioxidant response element (ARE) in the p62 promoter that is responsible for its induction by oxidative stress via NRF2 [5]. p62 is a stress-inducible protein that serves as an adaptor between selective autophagy and ubiquitine signaling. However, new evidence supports an autophagy-independent role for p62 as multifunctional signaling hub with many binding partners [6]. In particular, the two molecules NRF2 and p62 may engage in a crosstalk to sustain each other and increase chemoresistance [7]. Owing to its important role in protecting cells from cytotoxicity associated with reactive oxygen species (ROS) and electrophilic stressors [8], NRF2 can act as a tumor suppressor delaying carcinogenesis. On the other hand, NRF2 aberrant activation has been linked to cancer progression and chemoresistance, following, for instance, the NRF2-induced repression of apoptotic genes. Therefore, for these two opposite effects in cancerogenesis and tumor progression, NRF2 is considered a “double face” molecule with both tumor suppressive and tumor-promoting functions [9,10]. Hence, the targeting of the NRF2 pathway may represent a promising potential antineoplastic strategy, especially to increase cancer cell death-sensitivity [2,11].

We recently found that NRF2 activation, by a novel ruthenium(II)–curcumin compound, plays a pro-survival role in both wild-type (wt) p53- and mutant (mut) p53-carrying cancer cells balancing cell death/survival; thus, inhibiting the NRF2 survival pathway could increase cancer cell death [12,13]. Nonetheless, the curcumin compound was able to downregulate mutp53 (in mutp53-carrying cancer cell lines) through HSP90 downregulation and induce a certain degree of cell death, and such outcome was improved by NRF2 silencing [13]; in addition, the curcumin compound was able to activate wtp53 (in wild-type p53-carrying cancer cell lines), following DNA damage, and even in this case, the cell death outcome was increased after NRF2 silencing [13], suggesting that NRF2 silencing might improve the p53 response in anticancer therapies.

Tumor suppressor p53 *(TP53*) is the most important tumor suppressor gene, protecting cells from DNA damage and, therefore, from genomic instability that is responsible for the onset of cancer [14]. P53 is also a key molecule in the activation of the apoptotic pathways, for instance in response to anticancer drugs [15]. Therefore, a functional p53 pathway is mandatory for efficient cancer cell response to anticancer drugs. For these reasons, cancer cells cannot tolerate wtp53 that is, therefore, mostly inactivated in cancers by either gene mutations or protein deregulation [16]. In an attempt to re-establish wtp53 function, we previously found that the zinc–curcumin Zn(II)–curc compound can trigger mutp53 degradation, restoring p53-induced cancer cell death [13,17,18,19]. Mutant p53 proteins are often prone to loss of Zn(II) ion, which consequently promotes aggregation and, therefore, protein misfolding that results in a mutp53 that is more stable and resistant to proteasomal degradation [20]. In this regard, we found that zinc ions Zn(II) and zinc–curcumin compounds induce a conformational change in two of the most common p53 mutants, p53-R175H and -R273H, reestablishing the activity of the wtp53 allele that has been inhibited by the dominant-negative effect of the mutp53 allele [17,19,20,21]. We found that Zn(II) restores the wtp53 ability to induce the expression of the p53 target gene damage-regulated autophagy modulator (DRAM), a key regulator of autophagy, leading to autophagic induction that, in turn, triggers mutp53 degradation [18]. As for the wtp53, Zn(II) can improve wtp53 binding to target gene promoters and increase the effect of anticancer drugs, in combination therapy experiments [22,23,24,25,26]. Many preclinical studies have highlighted the benefit of phytochemical compounds as anticancer agents, especially in combination with conventional anticancer drugs [27]. However, the use of the zinc–curcumin complex as an antitumor metal-based drug can face some limitations, since curcumin has been shown to induce NRF2 [28], as also demonstrated by our previous findings [13,29], and to reduce cancer cell death. Indeed, NRF2 overexpression could play a cytoprotective role in cancer cells, regardless of p53 activation, which is one reason why the NRF2 pathway could be depicted as a molecular vulnerability to be therapeutically exploited. With this in mind, in the current study we aimed at evaluating if the Zn(II)–curc compound could induce NRF2 activation and the underlying molecular mechanisms, in an attempt to identify potential anticancer druggable targets that weaken the NRF2 pathway and restore cancer cell death sensitivity in cancers.

## 2. Materials and Methods

### 2.1. Cell Culture

Human breast cancer SKBR3 and glioblastoma U373 cell lines were grown, respectively, in Dulbecco’s modified Eagle’s medium (DMEM) and RPMI-1640 medium (Life Technologies-Invitrogen, Eggenstein, Germany), containing 10% heat-inactivated Foetal Bovine Serum (FBS) (Corning, NY, USA) at 37 °C and 5% CO_2_. They underwent routine testing to ensure that they were mycoplasm negative.

### 2.2. Antibodies and Reagents

The antibodies used for Western blotting analyses were mouse monoclonal anti-HO-1 (A-3, sc-136960), anti p62/SQSTM1 (D-3, sc-28359) and anti-Keap1 (G-2, sc-363626) from Santa Cruz Biotechnology (Dalla, TX, USA); rabbit polyclonal anti-NRF2 (ab62352) (Abcam, Cambridge, UK) l and mouse monoclonal β-actin (Calbiochem, San Diego, CA, USA). The reagents used were zinc–curcumin complexes Zn(II)–curc [30] dissolved in dimethyl sulfoxide (DMSO) and used as previously shown [17]; the NRF2 inhibitor Brusatol [31,32] (Sigma-Aldrich, Dorset, UK), used at 100 nM as previously reported [12]; autophagy inhibitor Bafilomycin A1 (BAF) (20 nM) (Sigma- Aldrich, Dorset, UK), added to the cell culture for the last 3 h, as previously reported [33]; and the endoplasmic reticulum (ER) stress inhibitor 4-Phenylbutyric acid (4-BPA) (Sigma-Aldrich, #P21005) [34], dissolved in filtered sterile water and used at 2.5 mM [35].

### 2.3. Western Blotting

Cells were lysed in lysis buffer (50 mM Tris–HCl, pH 7.5, 150 mM NaCl, 5 mM EDTA, 150 mM KCl, 1 mM dithiothreitol and 1% Nonidet P-40) (all from Sigma-Aldrich, Dorset, UK) containing protease inhibitors (Complete^TM^, Mini Protease Inhibitor Cocktail, Merck, Life Science S.r.l., Milan, Italy), sonicated and then centrifuged at 4 °C for 20 min. The supernatant protein lysates were separated by SDS-PAGE (polyacrylamide gel electrophoresis) gradient gels (Bio-Rad, Hercules, CA, USA), following semidry blotting to polyvinylidene difluoride (PVDF) membranes (Immobilon-P, Merk-Millipore, Milan, Italy). Membranes were blocked in Tris buffered saline containing 0.1% Tween 20 (TBS) and 3% BSA (Sigma-Aldrich, Dorset, UK) before probing with the primary antibodies and then with the appropriate secondary antibodies coupled to horseradish peroxidase (HRP) (Bio-Rad, Segrate, MI, Italy). Enzymatic signal was visualized by chemiluminescence (ECL Detection system, Amersham GE Healthcare, Milan, Italy).

### 2.4. siRNA Interference

Cells, plated at subconfluency in 35 mm Petri dishes, were transfected with the Nrf2 siRNA (sc-3703), sip62 (SQSTM1 siRNA, sc-29679) or control siRNA (sc-37,007), all from Santa Cruz Biotechnology using LipofectaminePLus reagent (#11514015, Thermo Fisher Scientific, Walthman, MA, USA), as previously reported [13,36].

### 2.5. Cell Viability Assays

Cells were plated in six-well plates using three to five replicates. The day after plating, cells were treated with Zn(II)–curc (100 μg/mL) for 16–24 h with or without pre-treatment with brusatol (100 nM for 4 h). After treatments, cells (both floating and adherent ones) were collected and stained with Trypan blue (Sigma-Aldrich, Dorset, UK) to assess cell viability counting blue (dead)/total cells with a Neubauer hemocytometer using light microscopy.

### 2.6. Chromatin Immunoprecipitation (ChIP) Assay

For Chromatin Immunoprecipitation (ChIP) analysis, protein/DNA complexes were cross-linked by adding formaldehyde (1% final concentration) in living cells, as previously reported [16], and chromatin extracts were incubated overnight at 4 °C with anti-NRF2 antibody (ab62352) (Abcam, Cambridge, UK). Then, protein G (Pierce), blocked with 1 μg/μL sheared herring sperm DNA and 1 μg/μL bovine serum albumin (BSA for 3 h at 4 °C), was incubated with chromatin and antibodies for 2 h at 4 °C. PCR was performed with Hot-Master Taq polymerase (Eppendorf, Milan, Italy) using immuniprecipitated DNA and promoter specific primers for human HO-1: HMOX1 For: CACGGTCCCGAGGTCTATT; Rev: TAGACCGTGACTCAGCGAAA. Immunoprecipitation with non-specific immunoglobulins (IgG; Santa Cruz Biotechnology, Dallas, TX, USA) was performed as negative controls. PCR products were run on 2% agarose gel. Densitometric analysis was performed to measure the amount of precipitated chromatin in each PCR and normalized with the input of each immunoprecipitation.

### 2.7. RNA Extraction and Semiquantitative Reverse Transcription (RT)-PCR Analysis

Total RNA extraction was performed by using TRIzol Reagent (Thermo Fisher Scientific, Walthman, MA, USA); cDNA was synthesized by using an MuLV reverse transcriptase kit (Applied Biosystems, Foster City, CA, USA); and semiquantitative Reverse-Transcribed (RT)-PCR was carried out by using Hot-Master Taq polymerase (Thermo Fisher Scientific, Walthman, MA, USA). Primer sequences are as follows: NRF2 For: TCCATTCCTGAGTTACAGTGTCT; Rev: TGGCTTCTGGACTTGGAACC. HO-1 For: AAGATTGCCCAGAAAGCCCTGGAC; Rev.: AACTGTCGCCACCAGAAAGCTGAG. p62 For: CTGCCCAGACTACGACTTGTGT; Rev.: TCAACTTCAATGCCCAGAGG. 28S For: GTTCACCCACTAATAGGGAACGTGA; Rev: GATTCTGACTTAGAGGCGTTCAGT. Densitometric analysis was performed to quantify mRNA levels compared to the control 28S gene expression.

### 2.8. Statistical Analysis

Statistical significance was determined using Student’s *t*-tests for two-sample comparison. Difference was considered statistically significant when the *p*-value was at least <0.05.

## 3. Results and Discussion

### 3.1. Induction of the NRF2 Pathway by Zn(II)–Curc

**Figure 1:**
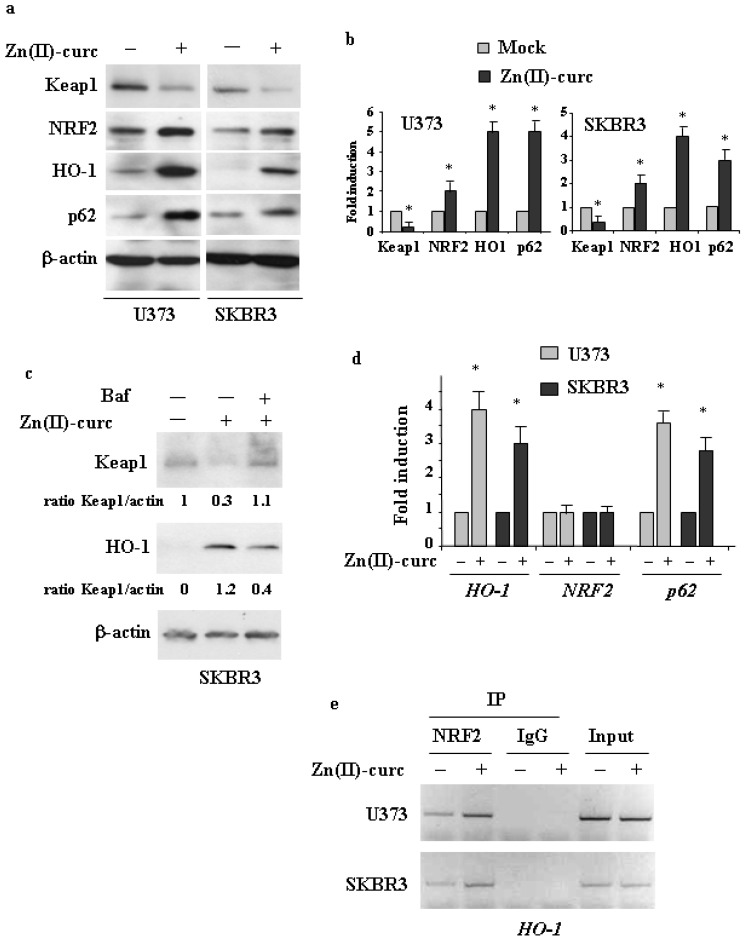
Zn (II)–curc activates NRF2. (**a**) Western blot analysis of Keap1, NRF2, HO-1 and p62 protein levels in U373 and SKBR3 cells untreated or treated with Zn (II)–curc (100 μg/mL) for 24 h. (**b**) Densitometric analysis was performed using Image J software to calculate the ratio of the protein levels, as detected in (**a**), vs. β-actin, with the control set to 1. Histograms represent the mean ± SD of three independent experiments. * *p* ≤ 0.05. (**c**) Western blot analysis of Keap1 and HOI-1 in SKBR3 cells undergoing Bafilomycin A1 (BAF) (20 nM) treatment for 3 h followed by Zn (II)–curc (100 μg/mL) for 16 h. Anti-β-actin was used as protein loading control. (**d**) Total mRNA was extracted from U373 and HT29 cells untreated or treated with Zn (II)–curc (100 μg/mL) for 16 h. *HO-1*, *Nrf2* and *p62* gene expression was assayed by the polymerase chain reaction (PCR) of reverse-transcribed cDNA. Densitometric analysis was performed using Image J software to calculate the gene expression/28S ratio. Histograms represent the mean ± SD of three independent experiments. * *p* ≤ 0.05. (**e**) U373 and SKBR3 were treated with Zn–curc (100 μg/mL) for 16 h before being assayed for chromatin immunoprecipitation analysis (ChIP) with anti-NRF2 antibody. PCR analysis was performed on the immunoprecipitated protein/DNA complex using primers specific for *HO-1* promoter. A sample representing linear amplification of the total chromatin (input) was included as control. Additional controls included immunoprecipitation performed with non-specific immunogloblulins (IP: IgG).

To investigate if the Zn(II)–curc compound could induce NRF2 activation, we used two different cancer cell lines, that is, U373 glioblastoma and SKBR3 breast cancer, both bearing mutp53, and against which we previously demonstrated the anticancer effects of Zn(II)–curc treatment [17,18,19,35]. These cell lines have two different p53 mutations, respectively, R273H and R175H, which are the most frequent p53 mutations in cancer patients and which have been shown to acquire oncogenic functions [16]. Given that mup53 often engages in a crosstalk with other oncogenic pathways [37], to understand how these pathways are regulated and then can be targeted is of relevance for the success of anticancer therapies. For this reason, the use of mutp53-carrying cancer cells can be of help. As shown in Figure 1a, Zn(II)–curc treatment greatly increased the NRF2 protein levels along with its targets HO-1 and p62, while markedly downregulated Keap1 levels, in both cell lines. These latter results are in agreement with the finding that p62 may trigger Keap1 degradation to induce NRF2 noncanonical activation [3]. As expected, the treatment with autophagy inhibitor bafilomycin A1 (BAF) abolished the Zn(II)–curc-induced Keap1 downregulation and thus counteracted HO-1 stabilization (Figure 1b). These results suggest that Zn(II)–curc promoted NRF2 activation by inducing Keap1 downregulation through autophagy, also because NRF2 mRNA was not upregulated (Figure 1c), strengthening that its regulation occurred at the protein level. In agreement with the activation of NRF2, its targets HO-1 and p62 were both induced at the mRNA level (Figure 1c). The NRF2 activity, assessed by ChIP assay, showed that the efficient NRF2 in vivo recruitment onto the HO-1 promoter in response to Zn(II)–curc indeed occurred (Figure 1d). These findings indicate that NRF2 was efficiently activated by Zn(II)–curc likely due to the activation of the p62/Keap1 pathway that contributes to NRF2 stabilization in a positive feedback loop. Thus, p62 is among the NRF2 targets [5,7], and its overexpression, in turn, favors NRF2 activation through Keap1 degradation [4].

### 3.2. The Interplay between NRF2 and p62 in Cancer Cells Treated by Zn(II)–Curc

**Figure 2:**
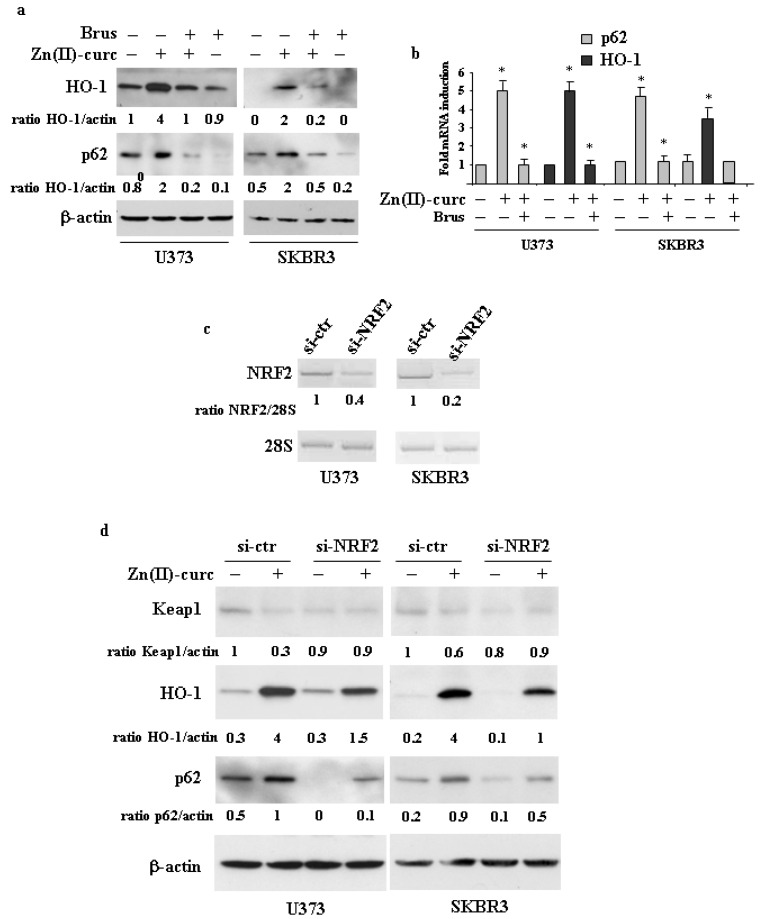
NRF2 silencing impairs p62 induction. (**a**) U373 and SKBR3 cells were pre-treated with brusatol (Brus, 100 nM for 4 h) and then treated with Zn–curc (100 μg/mL) for 24 h. Equal amounts of total cell extracts were analyzed by Western immunoblotting with anti-HO-1 and anti-p62 antibodies. Representative images are shown. Anti-β-actin was used as protein loading control. Densitometric analysis was applied to quantify HO-1 or p62/β-actin ratio. (**b**) U373 and SKBR3 cells were pre-treated with brusatol (Brus, 100 nM for 4 h) and then treated with Zn–curc (100 μg/mL) for 16 h. *HO-1* and *p62* gene expression was assayed by the polymerase chain reaction (PCR) of reverse-transcribed cDNA. Densitometric analysis was performed using Image J software to calculate the gene expression/28S ratio. Histograms represent the mean ± SD of three independent t experiments. * *p* ≤ 0.05. (**c**) U373 and SKBR3 cells were transfected with siRNA control (si-ctr) and siNRF2 and 36 h after transfection assayed for RT-PCR of *NRF2* gene expression. Densitometric analysis was performed using Image J software to calculate *NRF2* gene expression/28S ratio. (**d**) U373 and SKBR3 cells, transfected with siRNA control (si-ctr) and siNRF2 and 36 h, were then treated with Zn–curc (100 μg/mL) for 24 h. before Western blot analysis of the indicated proteins. Actin was used as protein loading control. The ratio of the proteins level vs. β-actin, following densitometric analysis using Image J software, is reported.

To investigate the interplay between NRF2 and p62 in cancer cells treated with Zn(II)–curc, we reduced NRF2 activation by pharmacologic means or translational silencing. To this aim, we first used brusatol, which is largely used as a pharmacologic NRF2 inhibitor [31,32], and found that it markedly impaired the Zn(II)–curc-induced p62 and HO-1 expression at both protein (Figure 2a) (shown as ratio of HO- or p62/actin underneath the gels) and mRNA levels (Figure 2b), strongly suggesting a reduction in NRF2 transcriptional activity. To assess the role of NRF2 more specifically, we performed NRF2 translational silencing with specific small interference RNA (siRNA) (Figure 2c). The results show that the Zn(II)–curc-induced increase in p62 and HO-1 expression was greatly counteracted by NRF2 knockdown (Figure 2d). Of note, Zn(II)–curc failed to induce Keap1 downregulation in cells undergoing NRF2 silencing (compared to si-ctr cells), in agreement with the impairment of p62 induction (Figure 2d), strengthening the interplay between p62/Keap1 and NRF2.

**Figure 3:**
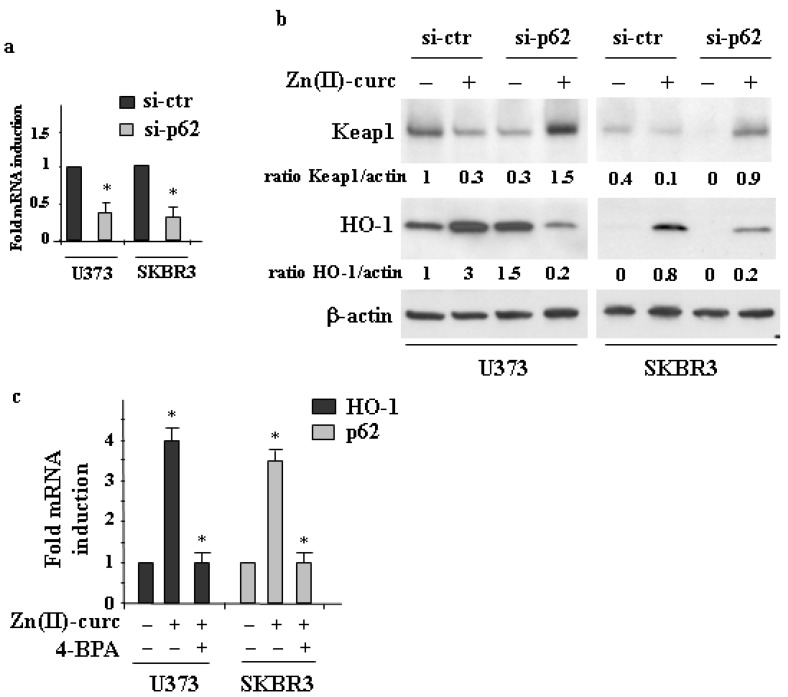
p62 silencing impairs NRF2 activation. (**a**) U373 and SKBR3 cells were transfected with siRNA control (si-ctr) and sip62 and 36 h after transfection assayed for RT-PCR of *p62* gene expression. Densitometric analysis was performed using Image J software to calculate *p62* gene expression/28S ratio. (**b**) U373 and SKBR3 cells, transfected with siRNA control (si-ctr) and sip62 and 36 h, were then treated with Zn–curc (100 μg/mL) for 24 h before Western blot analysis of the indicated proteins. Actin was used as protein loading control. The ratio of the proteins level vs. β-actin, following densitometric analysis using Image J software, is reported. (**c**) U373 and SKB3 cells were treated with Zn(II)–curc (100 μg/mL) for 24 h, with or without 1 h pre-treatment with 4-BPA (2.5 mM) before assaying for RT-PCR analyses of gene expression. Densitometric analysis was performed using Image J software to calculate the p62 or HO-1/28S ratio. Histograms represent the mean ± SD of three independent experiments. * *p* ≤ 0.05.

To further address the relationship between p62 and NRF2, we next performed p62 silencing with specific siRNA (Figure 3a). We found that p62 depletion strongly impaired the Zn(II)–curc-induced HO-1 protein levels (Figure 3b) and markedly counteracted the Zn(II)–curc-induced Keap1 downregulation, which was increased by such treatment (Figure 3b). These results are in agreement with the finding that p62 induces Keap1 degradation stabilizing NRF2 [38,39]. In order to further evaluate the mechanism of p62 induction, we took advantage of our previous findings, showing that Zn(II)–curc treatment induces endoplasmic reticulum (ER) stress [18,35] that is linked to NRF2 activation and further to p62 upregulation [40]. We found that the inhibition of ER stress by 4-BPA [34] impaired the Zn(II)–curc p62 as well as HO-1 expression, by RT-PCR analyses (Figure 3c).

Collectively, these results demonstrate that Zn(II)–curc-induced NRF2 and p62 sustained each other in a positive feedback loop.

### 3.3. Role of p62/NRF2 Axis in Zn(II)–Curc-Induced Cell Death

**Figure 4:**
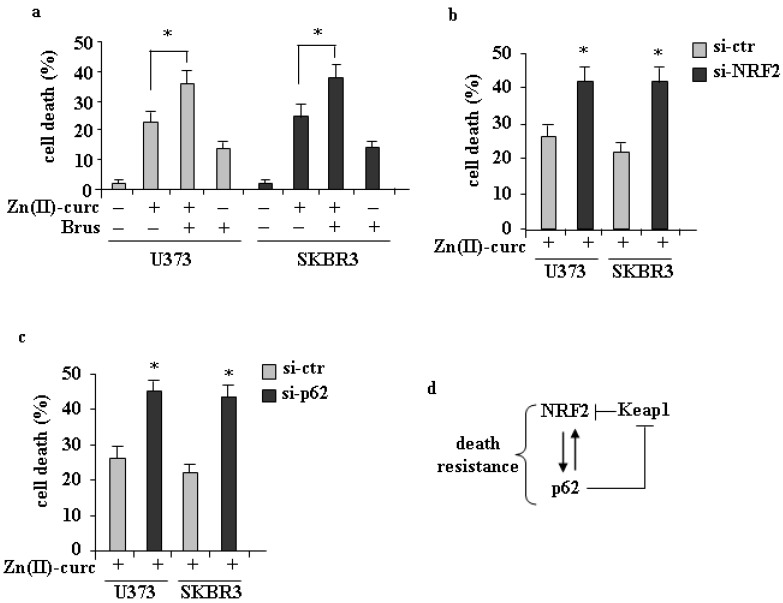
Inhibition of NRF2/p62 axis increases sensitivity to Zn–curc-induced cell death (**a**) U373 and SKBR3 cells were pre-treated with brusatol (Brus, 100 nM for 4 h) and then treated with Zn–curc (100 μg/mL) for 24 h. Cell viability was measured by trypan blue exclusion assay and expressed as percentage ± SD of three independent experiments. (**b**) U373 and SKBR3 cells, transfected with siRNA control (si-ctr) and siNrf2 or (**c**) sip62for 36 h, were then treated with Zn–curc (100 μg/mL) for 24 h before assaying cell viability as in (a). * *p* ≤ 0.05. (**d**) Schematic representation of the interplay between p62/Keap1/NRF2 and the biological outcome.

Both p62 and NRF2 have been demonstrated to play a prosurvival role in cancer cells [1,6,41]. We previously demonstrated that Zn(II)–curc can induce cell death through several mechanisms involving p53 function [17,18,19,20,21,22,23,24,35]. However, the findings of this study led us to hypothesize that the p62/NRF2 axis could hinder the cancer cell death sensitivity to Zn(II)–curc. To test this hypothesis, we inhibited NRF2 activity with brusatol or with specific siRNA. The results show that NRF2 inhibition or silencing significantly increased Zn(II)–curc-induced cell death (Figure 4a,b), in agreement with our hypothesis. Parallel experiments performed with p62 silencing using specific siRNA indicated that also p62 knockdown increased Zn(II)–curc-induced cell death (Figure 4d), in agreement with the p62/NRF2 interplay. These results demonstrate that the NRF2/p62 interplay, activated in response to Zn(II)–curc treatment, could act as a survival pathway and, therefore, represents a double sided potential anticancer druggable target.

## 4. Conclusions

In conclusion, we showed that the activation of the p62/Keap1/NRF2 pathway can play a pro-tumorigenic role lowering cancer cell sensitivity to death-inducing drugs such as Zn(II)–curc. The NRF2 signaling pathway is deregulated in many cancers by multiple mechanisms, including genetic, epigenetic and transcriptional changes. Increased NRF2 levels have been shown in many clinical cancer studies, including pancreatic, bladder, breast, glioma, gastric and colon [42,43,44,45,46,47,48,49,50,51,52]. Therefore, the modulation of the NRF2 pathway has attracted great attention for pharmacologic application in personalized medicine [11]. Among the molecules used to inhibit NRF2 signaling is brusatol, a natural quassinoid found to stimulate poly-ubiquitination of NRF2 [31,32]. Recently, the Aurora Kinase inhibitor AT9283 has been shown to have greater cytotoxicity in cells with NRF2 hyperactivation [51], highlighting the promising preclinical potential of this molecule in cancer overexpressing NRF2. Thus, the stimulation of NRF2 activity can reduce chemosensitivity in cancer cells [9,10]. In this regard, curcumin has been shown to induce the NRF2 pathway, which, while on one hand may be beneficial to halt cancerogenesis in its first stage, on the other hand can be detrimental when NRF2 defense mechanisms shield tumor cells from the effects of anticancer therapies. High p62 levels promote the activation of NRF2, which in turn induces the transcriptional activation of the p62/SQSTM1 gene, further increasing p62 accumulation through a feed-forward loop [4,38,39], and, therefore, p62 can be considered a key pro-oncogenic regulator [6] to be targeted in anti-cancer therapy. However, given their crosstalk, targeting p62 or NRF2 may influence each other and, consequently, both represent potential druggable targets in cancers with p62 and NRF2 overexpression to also improve the anti-cancer therapies through reactivation of the p53 oncosuppressor function [13,29].

## Data Availability

Not applicable.
